# The Story of the Insane from Year to Year

**Published:** 1901-02-16

**Authors:** 


### THE STORY OF THE INSANE FROM YEAR TO YEAR.

(THIRTEENTH SERIES.)

### THE KENT COUNTY ASYLUM AT MAIDSTONE.

THIS asylum contained 1,533 patients on January 1st, 1899, and the number increased by 20 during the year. There were 356 admissions, 36 re-admissions, 134 recoveries, 15 discharged relieved, and 211 deaths. The recoveries were 35·7 per cent. of the admissions, and the deaths were 13·6 per cent. of the average number resident. The former of these percentages is just a little below the average, while the latter is considerably over it. Of the deaths, 29 were due to cerebral and spinal diseases, 45 to phthisis, 23 to pneumonia, 8 to bronchitis, and 1 to enteritis. In 25 of the admissions the insanity was caused by such moral causes as domestic trouble, mental anxiety, &c., in 22 the cause was intemperance, in 90 previous attacks, and in 92 hereditary influence. Dr. Davies says "the general health of the asylum has been good," and he offers no explanation of the high mortality from lung diseases. The commissioners remark that phthisis accounted "for no less than 23 per cent. of the deaths," and they "draw attention to the overcrowding which is everywhere apparent." It would seem that close on 600 out-county cases are in residence, and that the contract for these will expire on the last day of 1900. The weekly cost of maintenance is a fraction under 10s. a head.

### THE BRISTOL ASYLUM.

THIS is the Asylum for the City and County of Bristol. On January 1st, 1899, it contained 773 patients, and on the last day of December there was an increase of 5 only. There were 198 first admissions, 39 readmissions, 90 recoveries and 129 deaths. Calculated on the admissions the recoveries stand at 37·97 per cent., and calculated on the average daily number resident the deaths stand at 16·37 per cent. These percentages show that the recoveries were slightly over the Asylum average and the deaths much over it and also over the average of the Bristol Asylum. Of these deaths, cerebral and spinal diseases account for 41, phthisis for 8, pneumonia for 19, pleurisy for 3, and bronchitis for 5. As to the causes of insanity in the year's admissions, it would seem that only three were due to moral causes as distinct from physical causes, and that among the latter drink is credited with the cause in 25 and hereditary influence in 81. General paralysis is put down as the cause in 19 cases, but as this is a form of insanity it is the cause of the general paralysis which ought to be stated. Dr. Benham points out that there has been a steady rise in the number of the admissions during the last five years, and that in 1895 there were only 136, whereas in 1899 they were 212; and it is further shown that, owing to the extension of the Borough boundaries, there are 45 patients chargeable to Bristol, but now in the Gloucester County Asylum. As a set-off against these figures there are 32 out-county patients at present in the asylum; and additions are being made to the building. There were two cases of enteric fever, but neither Dr. Davis, the Medical Officer of Health, nor Dr. Benham could find any cause for the disease, and the presumption is that it was introduced from without. Probably this was the case; but, as we have before remarked in these notices, sporadic cases of a disease apparently identical with enteric fever are occasionally seen in asylums; and such disease seems to have no tendency to spread. Instructions to the staff are continued, and many of them hold the nursing certificate of the Medico-Psychological Association, and have gone through the various grades of the St. John's Ambulance Society. These are admirable things; but they do not comprise a three years' course, and we should much like to hear that Dr. Benham has instituted a complete course of training. The committee express "their high appreciation of the manner in which the medical superintendent and the officers and staff of the asylum have performed the duties entrusted to them." The weekly charge for borough patients is 10s. 6d. each, while private patients pay 20s., and out-county cases from 14s. to 25s.

### THE ROYAL ASYLUM, ABERDEEN.

ON January 1st, 1899, this asylum contained 840 patients and at the end of the year 836 were in residence, 240 being private patients and 596 paupers. There were 204 first admissions, 60 readmissions, 130 recoveries, 31 discharged relieved, and 78 deaths. The recoveries stand at the high percentage of 49·2 of the admissions, and the deaths at 9·3 on the average number resident. Of the deaths 22 were caused by cerebral and spinal diseases, 15 by phthisis, and 16 by other lung diseases. Of the year's admissions the insanity was caused by moral causes in 9 cases, by intemperance in drink in 33, and by hereditary influence in 76. Dr. Reid chronicles an outbreak of enteric fever. Three of the staff in the laundry and two patients were affected, and they all recovered. The disease was introduced by a new patient in a state of acute excitement. A fire broke out in the old laundry, and the whole structure was destroyed in an hour. Fortunately the new laundry was ready for occupation. Dr. Matthews has given instructions to the staff on the subjects connected with the nursing of the insane, and 13 went up for the examination of the Medico-Psychological Certificate, and all the candidates passed. In the main building private patients pay from £30 to £50 a year. In Elmhill House the charges are from £60 to £250. The charge for paupers is £32 a year.

